# Molecular analysis reveals heterogeneity of mouse mammary tumors conditionally mutant for Brca1

**DOI:** 10.1186/1476-4598-7-29

**Published:** 2008-04-07

**Authors:** Mollie H Wright, Ana I Robles, Jason I Herschkowitz, Melinda G Hollingshead, Miriam R Anver, Charles M Perou, Lyuba Varticovski

**Affiliations:** 1 Center for Cancer Research, National Cancer Institute, 9000 Rockville Pike, Bethesda, MD 20892, USA; 2 Developmental Therapeutics Program, Division of Cancer Treatment and Diagnosis, FVC/205, Frederick, MD 21701, USA; 3 Lineberger Comprehensive Cancer Center, University of North Carolina, Chapel Hill, NC 27599, USA; 4 Pathology, SAIC Frederick, Inc., NCI-Frederick, FVC/301, Frederick, MD 21702, USA

## Abstract

**Background:**

Development of therapies for patients with BRCA1 mutations has been hampered by lack of readily available *in vitro *and *in vivo *models. We recently showed that transplantation of transgenic mammary tumors as cell suspensions into naïve recipients generates reproducible tumors with remarkable stability of gene expression profile. We examined the expression profiles of original and serially transplanted mammary tumors from *Brca1 *deficient mice, and tumor derived cell lines to validate their use for preclinical testing and studies of tumor biology.

**Methods:**

Original tumors, serially transplanted and multiple cell lines derived from *Brca1 *mammary tumors were characterized by morphology, gene and protein expression, and cell surface markers.

**Results:**

Gene expression among *Brca1 *tumors showed more heterogeneity than among previously characterized tumors from MMTV-*PyMT *and -*Wnt1 *models. Gene expression data segregated *Brca1 *tumors into 3 distinct types: basal, mixed luminal, and tumors with epithelial-to-mesenchymal transition (EMT). Serial transplantation of individual tumors and multiple cell lines derived from the original tumors recapitulated the molecular characteristics of each tumor of origin. One tumor had distinct features of EMT and gave rise to cell lines that contained a distinct CD44^+^/CD24^-/low ^population that may correlate with human breast cancer stem cells.

**Conclusion:**

Although individual tumors expanded by transplantation maintain the genomic profile of the original tumors, the heterogeneity among *Brca1 *tumors limits the extent of their use for preclinical testing. However, cell lines offer a robust material for understanding tumor biology and response to therapies driven by BRCA1 deficiency.

## Introduction

Breast cancer is the most common neoplastic disease in women and affects approximately 1 out of 10 females. Human breast cancer is a heterogeneous disease with varied clinical course [1,2]. Up to 5% breast cancer cases are attributable to inherited mutations in the *BRCA1 *or *BRCA2 *genes [3]. Inherited mutations of *BRCA1 *(chromosome 17q21) have been linked to increased risk for breast and ovarian cancers [4]. The BRCA1 tumor suppressor plays a major role in DNA damage, signaling, repair and cell cycle control. BRCA1 is also a co-regulator of steroid hormone receptors and modifies steroid hormone action [5]. Carriers of the mutant gene also have significantly higher risk for developing other tumor types, including ovarian, uterine, cervical, and prostate cancers [6]. Breast cancer patients with *BRCA1 *mutations are more likely to be estrogen receptor (ER) and HER-2 negative, and have mutant or deleted p53 [7]. In spite of advances in detection and clinical management for patients with familiar BRCA1 mutant breast cancer, there has been no significant improvement in therapies and overall survival for these patients [8].

Microarray analysis has been useful in identifying gene expression signatures that characterize qualities important for biological and clinical classification [9,10]. Phenotypic characterization and microarray profiling of breast tumors reveals distinct subtypes of breast carcinoma that are associated with survival. Five major groups of invasive breast carcinomas have been identified: luminal A, luminal B, HER2 +/ ER -, basal-like, and normal breast-like [10]. The basal-like tumors are typically ER and HER2-negative, have high proliferative rate, and typically show a poor clinical outcome [10].

BRCA1 and BRCA2 are essential for maintenance of genomic integrity by promoting repair of double-stranded DNA breaks. Following DNA damage, BRCA1 is phosphorylated by the ATM/ATR kinases and recruits multiple factors to the break site that actively participate in repair [11]. BRCA1 associates and co-localizes with RAD51 nuclear foci in mitotic cells along with BRCA2 and BARD1, a BRCA1 binding protein [12]. BRCA1 deficiency leads to impaired double-strand break repair and to enhanced sensitivity to ionizing radiation and genomic instability [11,13]. However, only limited studies are reported using human cells, most of them derived from analysis of one human cell line that is null for BRCA1, HCC1937 [14]. Although BRCA1 replacement increased the resistance to Vinorelbine and Cisplatin, it did not change sensitivity to other agents, such as Docetaxel [14] suggesting that multiple mechanisms may be associated with drug resistance in this cell line.

Studies of tumor biology and development of novel therapies for tumors associated with BRCA1 deficiency are hampered by the lack of readily available material for *in vitro *and *in vivo *studies. Genetically engineered mouse tumors are an excellent tool for studying cancer biology and can potentially improve preclinical studies. Development of multiple mouse models with deletion or mutations in *Brca1 *targeted to the mammary gland has provided an opportunity to examine the biology and therapeutic implications of BRCA1 loss in breast cancer [15] and some studies find similarities between the mouse and human tumors [11,16,17]. In particular, correlations with human basal-like tumors have been reported in *Brca1 *mouse models [17,18]. However, using *Brca1 *mouse mammary models for preclinical development has been limited due to complicated breeding schemes, variable penetrance, and prolonged latency of tumor development [19]. In spite of increased penetrance when mice heterozygous for the T*p53 *tumor suppressor are crossed with mice harboring mutant *Brca1*, these mice develop mammary tumors in over one year, at which time development of lymphomas, which is characteristic of p53-deficient background, also compromises animal survival [20].

Expansion of *Brca1 *deficient mammary tumors by transplantation is a useful alternative for generating sufficient material for studying *Brca1*-associated tumorigenesis. The purpose of our study was to harvest, expand *in vivo*, and characterize tumors and cell lines derived from *Brca1 *mammary tumors. The original and transplanted tumors as well as well-characterized cell lines derived from original tumors provide the necessary tools for studying the biology of *Brca1*-deficient tumors, identification of putative cancer stem cells, and development of novel therapies to improve clinical outcome.

## Materials and methods

### Tumors and serial transplantations of cell suspensions

All studies were conducted in an AAALAC accredited facility in compliance with the PHS *Guidelines for the Care and Use of Animals in Research*. Naïve 6-to-8 week old female *scid*/NCr (BALBc) mice from the NCI Animal Production Program (Frederick, MD) were used as transplant recipients. Autoclaved feed and hyper-chlorinated water were provided *ad libitum*. The *Brca1*^*Co*/*Co*Δ*exon*11^, *p53*^+/-^, *MMTV-Cre *mouse mammary tumors [21] were dissected and cell suspensions prepared as described by Varticovski and coworkers [22]. In summary, tumors were excised, dissected, mechanically dissociated and forced through a 40 uM mesh. Viable cells were either frozen in freeze down media, or plated at low density in 100 or 150 mm plates in RPMI supplemented with Pen/Strep, glutamine, and 2% FCS for selection of cell lines. Non-adherent cells were removed after 48 hours and surviving clones were isolated using cloning cylinders. For implantation of cell suspensions into naïve recipients, the mouse fat pad #4 of SCID mice was visualized through a small skin incision just anterior to the rear leg, and one million cells were injected in 50μ l of RPMI-1640. The incision was closed with a sterile wound clip, which was removed in 7 days.

Tumor size was determined biweekly using caliper measurements (millimeters) in two perpendicular dimensions (length and width). Tumor weights (milligrams) were calculated using the formula for a prolate ellipsoid and assuming a specific gravity of 1.0 g/cm^3 ^[23]. Mice were killed when tumors reached an approximate 1 g of wet weight. Tumors were divided into the following fragments: a portion was used to prepare cell suspensions and further passages *in vivo*, the remaining was frozen or fixed in 10% neutral-buffered formalin.

### Generation and genotyping of *Brca1 *tumor cell lines

Cells derived from *Brca1 *mammary tumors were grown at 37°C in 5% CO_2 _in RPMI-1640 media supplemented with increasing concentrations of FBS up to 10%, pen/strep, and glutamine. Over 40 clones were isolated. Sixteen cell lines were developed from 5 original primary tumors and maintained in culture for up to 50 passages in RPMI1640 supplemented with 10%FCS.

*Brca1 *primary tumors and cell lines were genotyped as described by PCR amplification of sequences specific for the null *Brca1 *allele [20], conditional *Brca1 *(Brca1^Co/Co^) allele [13,21], wild-type p53 allele [20], and CRE [20,24].

### RNA isolation, microarray hybridization and data analysis

Total RNA was isolated from 5 spontaneous (original, 0) *Brca1 *mammary tumors, 4 tumors from first-passage transplantation into naïve recipients (first-passage, 1), 3 tumors from second-passage transplantation into naïve recipients (second-passage, 2), and 7 representative original tumor-derived cell lines. Normal virgin mammary glands from three #4 glands of 6–8 wk old C57Bl6 or SCID mice were included in the microarray analysis. Total RNA was isolated using TRIzol reagent (Invitrogen, Carlsbad, CA) according to the manufacturer's instructions, followed by DNAse treatment and RNA clean-up (RNeasy Mini Kit, QIAGEN, Valencia, CA). RNA integrity was determined using the RNA 6000 Nano LabChip Kit on Agilent 2100 Bioanalyzer (Agilent, Santa Clara, CA). RNA was amplified and hybridized overnight to the Agilent Mouse Oligo Microarrays 22 K (G4121AorB) against a common reference total RNA [17]. The co-hybridized samples and reference RNA were washed and scanned on an Axon GenePix 4000B scanner (Molecular Devices, Sunnyvale, CA), analyzed using GenePix 4.1 software, and uploaded to the UNC microarray database where Lowess normalization was performed [25]. Genes were filtered by requiring the Lowess-normalized intensity values in both channels to be > 30. The log_2 _ratio of Cy5/Cy3 was then reported for each gene. Hierarchical clustering and Gene Set Expression Comparison were performed using BRB Array Tools software developed by the Biometric Research Branch of the NCI [26]. Analysis was restricted to probes that reported values in 75% or more of the samples for comparative analysis or probes that in addition had a minimum 2.5 – fold expression change in either direction from the median value in at least 20% of samples, for hierarchical clustering (defined as most variable genes).

A comparative analysis of previously published microarray expression data of Brca1-deficient tumors on the same array platform [17] was performed using Gene Cluster 3.0. To this end, we compared gene expression from 22 arrays encompassing the 5 original *Brca1 *tumors from our study, with 7 tumors from *Brca1*^+/-^, *p53*^+/- ^irradiated (IR, Kohler) mice, and 10 tumors from *Brca1*^*Co*/*Co*^, *p53*^+/-^, *MMTV-Cre *(Furth) from a previous study [17]. Genes [20,988] were filtered for 80% present (to remove genes that have missing values in greater than 20% of the arrays) and Standard Deviation (SD Gene Vector) >= 1.5 (to remove genes that have standard deviations of observed values less than 1.5, thus selecting for more variable genes across experiments). This filter yielded 339 genes that were used for Hierarchical Clustering. The dendrograms were visualized with Java TreeView.

### Analysis of cell surface markers

Cells from each cell line were grown to 70% confluence, scraped or trypsinized, stained with PE Anti-mouse CD44 (BD Pharmingen, San Jose, CA), APC Anti-mouse CD24 (Biolegend, San Diego, CA), FITC Anti-mouse CD44 (Southern Biotech, Birmingham, AL), or APC Anti-mouse CD117 (Biolegend). Rat IgG (CHEMICON, Billerica, MA) was used as the isotype control according to manufacturer's instructions. For analysis of Cytokeratins, cells were washed in ice-cold PBS, fixed in 70% ethanol, and stained with either Cytokeratin 5 (Covance, Berkeley, CA) or Cytokeratin 18 (Abcam, Cambridge, MA), and counter-stained with Alexa 488-conjugated anti-rabbit or anti-mouse IgG, respectively. Cells were analyzed by flow cytometry using FACScalibur or LSR II (BD Biosciences, San Jose, CA). Data were collected with Cell Quest Pro software (BD) from no fewer than 10,000 cells and FACSDiVa software (BD) from no fewer than 30,000 cells, respectively.

### Immunohistochemistry

Paraffin sections of original and transplanted tumors were stained for H&E for histological analysis. Histology of original tumors was compared to transplanted tumors derived from cell suspensions. Immunofluorescent staining was performed for smooth muscle actin (SMA) using mouse A2537 antibody (Sigma, St Louis, MO) at 1:1000 dilution and vimentin, using guinea pig anti-Vimentin antibody (RDI, Concord, MA) at 1:400 dilution. Slides were de-paraffinized and hydrated through a series of xylenes and graded ethanol steps. Heat-mediated epitope retrieval was performed in boiling citrate buffer (pH 6.0) for 15 min. Samples were then cooled to room temperature for 30 min. Secondary antibodies for immunofluorescence were conjugated with Alexa Fluor-488 or -594 fluorophores (1:200, Molecular Probes, Invitrogen, Carlsbad, CA).

Cell lines were plated in chamber glass slides, allowed to attach overnight, stained with APC Anti-mouse CD117 (c-kit) from Biolegend, Cytokeratins 5 and 18, and counter stained with Alexa 488-conjugated anti-rabbit or anti-mouse IgG, respectively.

### Immunoblot analysis

The analysis of protein levels was performed essentially as described [27]. Membranes were blocked at room temperature for 1 h I nTBS/0.05% Tween-20 (TBS-T) containing 5% non-fat dry milk (monoclonal antibodies) or 5% BSA (polyclonal antibodies) and exposed to primary antibodies overnight at 4°C in blocking solution. The following commercial antibodies were used: Keratin 5 (Covance); Keratin 18 (Abcam); CD117 (Abgent; San Diego, CA), p53(S15), PDGF Receptor (Anti-CD140a) (Chemicon), Vimentin (BD Pharmingen), and Actin (Ab-1) (Oncogene Research Products, Cambridge, MA). Membranes were washed twice in TBS-T and incubated with species-specific HRP-labeled secondary antibodies in TBS-T for 1 h. Finally, the membranes were washed in TBS-T three times and the proteins were visualized using ECL Western blotting detection reagents (Amersham, Piscataway, NJ).

## Results

### Gene expression analysis of *Brca1 *mammary tumors

**Figure 1:**
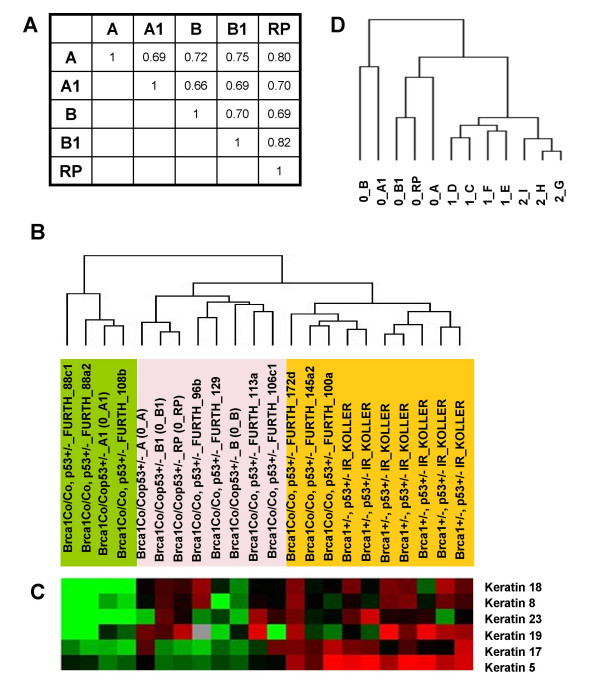
**Characterization of *Brca1 *tumors **(A) **Pearson correlation coefficient matrix for pairwise comparisons of log-ratios among five spontaneous *Brca1 *tumors, based on 15,781 probes with reported values in at least 75% of samples (missing-value filter).(B) **Unsupervised Hierarchical Cluster of 5 *Brca1*, 7 *Brca1*^+/-^, *p53*^+/- ^IR and 10 *Brca1*^*Co*/*Co*^, *p53*^+/-^, *MMTV-Cre *(Furth) mammary tumors based on 339 genes filtered for present values and minimal variability of expression (see Materials and Methods). **(C) **The dendrogram corresponds to the summarized data for expression of keratins 5, 8,17,18,19 and 23, that were among the 339 genes. The color of each cell represents the relative expression level of each gene.**(D) **Unsupervised Hierarchical Cluster of original (0), first- (1) and second- (2) passage tumors based on the 416 probes that in addition to passing intensity and missing-value filters had a minimum 2.5-fold expression change in either direction from the median value in at least 20% of samples (Additional file 1).

The overall similarity of gene expression profiles among five original *Brca1 *mammary tumors was assessed by pairwise correlations of microarray log-ratios based on 15,781 probes with reported values in at least 75% of samples. The correlation matrix showed Pearson correlation coefficients for pairwise comparisons of 0.66–0.82 (Figure 1A). These correlation coefficients are lower than those obtained for mouse mammary tumors from MMTV-PyMT (0.79–0.94) or MMTV-Wnt1 (0.87–0.95) mouse tumors using a similar analysis [22] (Additional file 1A). In spite of having a similar size at the time of collection (approximately 1 gm of wet weight), heterogeneity of *Brca1 *mammary tumors was also evident upon pathological examination of morphology, immunohistochemistry, and immunofluorescence analysis of tumor markers. Although all tumors were adenocarcinomas, the original tumor 0_A1 had areas of whorls and clusters of spindle-shaped tumor cells in a myxomatous stroma, features suggestive of epithelial-to-mesenchymal transition (EMT) (Additional file 2A). Consistently, this tumor was also positive for protein expression of a mesenchymal marker, vimentin (Additional file 2B). No evidence of EMT was found in any of the four other original tumors. One of the other tumors was an adenocarcinoma with features of squamous carcinoma (Additional file 2A).

### Comparison of *Brca1 *tumor expression profiles with other previously reported Brca1-deficient mouse mammary models

In previous studies [17,28] Brca1 loss was found to give rise to mammary tumors with a primarily basal-like phenotype. Two models with Brca1 deficiency were featured in a large study that used multiple other mouse mammary tumor models [17], and included 7 mammary tumors that arose after irradiation of *Brca1*^+/-^, *p53*^+/- ^mice (designated *Brca1*^+/-^, *p53*^+/- ^IR-Koller) and 10 tumors from *Brca1*^*Co*/*Co*^, *p53*^+/-^, *MMTV-Cre *strain, equivalent to the tumors used in our study (designated *Brca1*-Furth). Since the same array platform and common reference RNA had been used for those studies, we performed an analysis of all 22 samples as described in Methods and found 339 that distinguished these samples. Unsupervised Hierarchical Clustering using these 339 most variable genes (Additional file 3) separated the tumors in 3 main clusters (Figure 1B). All seven *Brca1*^+/-^, *p53*^+/- ^IR-Koller and 3/10 *Brca1*-Furth tumors showed a basal-type phenotype, previously segregated in Group IV, and characterized by expression of Keratin 5 [17]. Four of the original *Brca1 *tumors from our study clustered with those four *Brca1*-Furth tumors that had mixed basal and luminal features. An additional cluster was formed by the original *Brca1 *tumor A1 from our study and three of *Brca1*-Furth tumors previously segregated in Group II tumors of spindloid morphology [17]. This cluster showed mesenchymal-like features that included lack of epithelial markers, such as keratins. Moreover, the pattern of expression of keratins across all 339 filtered genes further defined separation of all 22 tumors into the same 3 groups (Figure 1C). Thus, although the majority of Brca1-deficient tumors are basal-like, some have mixed basal and luminal characteristics, and a third group has features consistent with EMT.

### Serial transplantation generates tumors with a stable gene expression profile

Given the apparent heterogeneity of *Brca1 *mammary tumors, we examined the extent to which serial transplantations of individual tumors affect gene expression. Cell suspension from one original (0_A) tumor was transplanted into naïve recipients. Unsupervised Hierarchical Clustering of 5 original *Brca1 *mammary tumors (0), 4 first-passage (1) and 3 second-passage (2) tumors from 0_A tumor were performed based on 416 most variable probes (Additional file 4). This analysis showed that the transplanted tumors segregated into a single branch closer to the tumor of origin, and further subdivided into two groups according to passage number (Figure 1D). As suggested by the Pearson correlation analysis, evidence that all original tumors were heterogeneous was indicated by the long distance of each sample from the immediate tree node above. Gene expression differences between 0_A tumor and the average of gene expression in all transplanted tumors showed 76 upregulated and 79 downregulated genes whose expression changed at least 3-fold (Additional file 4). However, comparison of gene expression between the first and second passage tumors showed no genes with significantly different expression (p = 0.2). Thus, serial transplantation of *Brca1 *mammary tumors resulted in minimal gene expression profile changes.

Because the *Brca1 *original tumors used for our studies were generated in mice bred in a mixed strain background composed mostly of C57Bl6 [21], and cell suspensions were transplanted into immunosuppressed SCID mice, we hypothesized that some of the genetic differences between the original and transplanted tumors could be associated with differences in the genetic background. Thus, we re-analyzed tumor samples with normal virgin mammary glands from C57Bl6 and SCID mice using the 416 most variable probes defined above (Additional file 5). This analysis revealed that several immune response genes, including Histocompatibility 2, class II antigen A, alpha (H2-aA), Histocompatibility 2, class II, locus Mb1 (H2-DMb1) and Histocompatibility 2, class II antigen A, beta 1 (H2-Ab1), as well as Lymphotoxin B (Ltb), and Interleukin 15 (Il15) were downregulated in both, the transplants and normal SCID mammary gland as compared to original tumors and normal C57Bl mammary gland. These data are consistent with immunodeficient genotype of SCID mice, and confirms that many of the differences in gene expression between the original and transplanted tumors are due to differences in genetic background.

The final analysis between the original and transplanted tumors showed that several of the upregulated genes in the transplanted tumors were associated with growth, metastasis, and cancer stem cells, such as Wnt inhibitory factor 1 (Wif1), Notch gene homolog 4 (Notch4), Hormonally-regulated Neu-associated kinase (Hunk), Sprouty homolog 1 (Spry1), Twist gene homolog 1 (Twist1), and Aldehyde dehydrogenase 3 family, member B1 (Aldh3b1). In addition, apoptosis-associated Phorbol-12-myristate-13-acetate-induced protein 1 (Pmaip1) was among the genes downregulated in transplanted tumors. These data are consistent with the accelerated growth observed in serially transplanted tumors [22].

### Expression profiling of cell lines and comparative analysis with the tumors of origin

**Figure 2:**
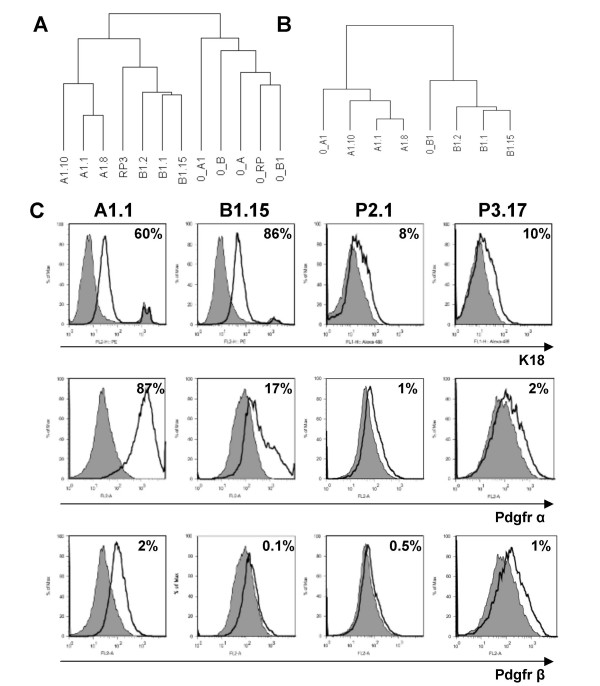
**Characterization of *Brca1 *cell lines **(A) **Unsupervised Hierarchical Cluster of original tumors (0) and cell lines using the 1660 probes that in addition to passing intensity and missing-value filters had a minimum 2.5-fold expression change in either direction from the median value in at least 20% of samples (most variable genes)**. **(B) **Unsupervised Hierarchical Cluster of the original tumors 0_A1 and 0_B1 along with the cell lines derived from them. Cluster was based on the 914 genes reporting at least 4-fold difference in expression ratios between original tumor 0_A1 vs original tumor 0_B1. **(C) **Cell lines were stained with specific fluorescently-conjugated mAb to Cytokeratin 18, Platelet-derived growth factor receptor (Pdgfr) α or β, as indicated, in parallel with appropriate isotype control-matched mAb and analyzed by flow cytometry. In the histograms shown, the thick black line represents positive staining and the gray filled lines represent the isotype control-matched staining. The percent of positive cells after compensation is shown on the upper right of each panel.

The differences between individual *Brca1 *tumors prompted us to generate and characterize multiple cell lines from each original mammary tumor. To ensure that all 16 cell lines derived from 5 original tumors were free of non-mammary cellular components, we genotyped these cells for *Brca1*^*Co *^and *p53 *alleles. We confirmed that all cell lines contained the recombined *Brca1*^*Co *^allele and, as shown for primary mammary tumors in this model, lost the wild type *p53 *allele [20] (Additional file 6). Microarray expression analysis of 7 representative cell lines from 3 original *Brca1 *tumors were compared to the individual tumors of origin. Cell lines B1.1, B1.2, and B1.15 were generated from the original tumor 0_B1, and the cell line RP3 was derived from 0_RP tumor. Cell lines A1.1, A1.8 and A1.10 were derived from the original tumor 0_A1. This analysis revealed 1,660 most variable genes. Unsupervised Hierarchical Clustering of all 12 samples using these 1,660 genes showed that the cell lines were discriminated from the original tumors (Figure 2A). However, all cell lines derived from the same tumor clustered together in the same sub-branch of the tree, indicating that molecular features of the original tumor are preserved in each set of cell lines derived from that tumor. Some of the differences in gene expression between original tumors and cell lines included downregulation of c-kit (CD117) and further downregulation Cytokeratins 23, 19, 18, 14, 13 and 8, in cell lines. Vimentin, Platelet-Derived Growth Factor receptor alpha (Pdgfr α) and beta (Pdgfr β) were among genes that were upregulated in cell lines consistent with adaptation to tissue culture conditions.

To further assess the conservation of features from original tumors to cell lines, we applied the Gene Set Expression Comparison tool. This tool analyzes pre-defined gene sets for differential expression among pre-defined classes, and indicates which gene set(s) contain more differentially expressed genes than expected by chance. A gene set representing differences in gene expression between the two original tumors 0_A1 and 0_B1 was generated that contained 914 probes either up- or down-regulated by at least 4-fold. This gene set was enriched in genes that were also differentially expressed among the cell lines, with high significance (p < 1e^-07^). Consistently, Unsupervised Hierarchical Clustering of those original tumors and cell lines based on the 914 differentially expressed probes showed that the cell lines segregated with each tumor of origin (Figure 2B). Genes with differences in expression that were maintained in the original 0_A1 tumor and in cell lines derived from this tumor as compared to 0_B1 tumor and its respective cell lines included loss of CD24a, indicating a possible enrichment in putative cancer stem cells [29,30]. These data indicate that although the original tumors are heterogeneous, the groups of cell lines derived from each one of them are similarly different from each other.

### Correlation of gene expression profile with protein expression

We examined the expression of several gene products in all cell lines by Flow Cytometry and Western Blot. Although Cytokeratin 18 was downregulated in all cell lines by expression profiling, the protein was still detectable by FACS (Figure 2C) and Western Blot (Additional file 7). Upregulation of Pdgfr α which was detected by microarray analysis, was confirmed by FACS (Figure 2C) and Western blot (Additional file 7), while only a low level of Pdgfr β protein was detected in some cell lines. Variable expression of Vimentin and CD117 (c-kit) and a consistent loss of Cytokeratin 5 in all cell lines was observed by Western blot analysis (Additional file 7) which correlated with gene expression. Interestingly, cell lines derived from anterior and posterior tumors differed in expression of CD117 (c-kit) with protein expression detected only in cell lines derived from tumors arising in posterior mammary glands (Additional file 8).

### Detection of putative cancer stem cell markers in *Brca1 *cell lines

**Figure 3:**
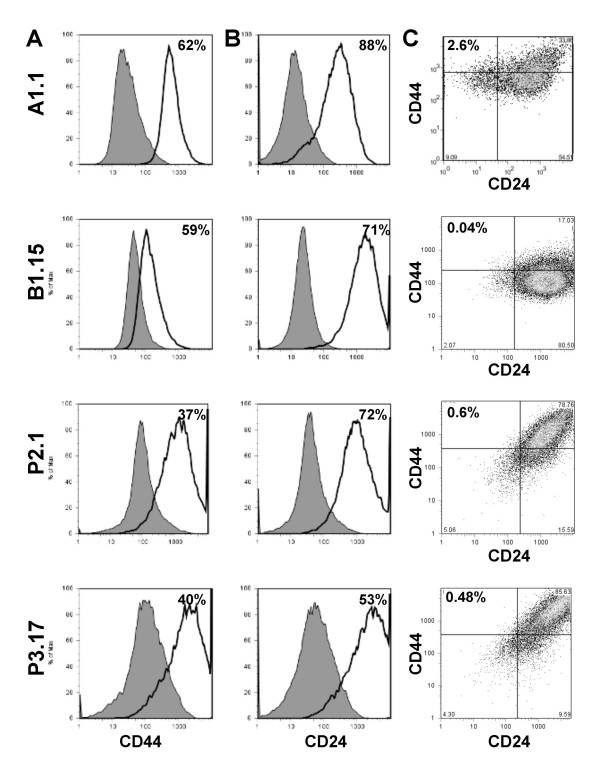
**Characterization of cancer stem cell markers in *Brca1 *cell lines. **Cell lines A1.1, B1.15, P2.1 and P3.17 were stained with fluorescently-conjugated mAb specific to CD44 **(A)**, or CD24 **(B)**, in parallel with isotype control-matched mAb and analyzed by flow cytometry. In the histograms shown, the thick black line represents positive staining and the gray filled lines represent the isotype control-matched mAb. **(C) **Bivariate plots represent the double staining of cell lines with fluorescently-conjugated CD44 and CD24 mAb. The quadrants are gated based on isotype controls and separate the double positive, single positive, and double negative populations. The percent of positive fraction for single label and CD44^+^/CD24^- ^cells after compensation are indicated in each panel.

Recent studies identified putative human breast cancer stem cells by CD44^+^/CD24^low ^cell surface markers that correspond to normal mammary gland-initiating cells in the mouse model [29,30]. Because we found a decrease in CD24a gene expression in the original 0_A1 tumor and in all cell lines derived from this tumor, we examined whether these cells contain a distinct CD44+/24^-/low ^population. We performed flow cytometry on multiple cell lines representative of 0_A1 and all 5 original tumors using CD44 and CD24 markers (Figure 3A). Remarkably, the A1.1 cell line which is derived from 0_A1 tumor showed up to 2.6% CD44^+^/CD24^-/low ^cells. Analysis of additional cell lines showed that all cell lines derived from 0_A1 tumor were enriched in CD44^+^/CD24^-/low ^population with 1.32–5% (Figure 3A and data not shown). In contrast, this cell population was less than 1% in all other cell lines derived from other tumors (Figure 3C). Specifically, B1.15 had 0.39%, P2.1 0.60%, and P3.17 0.46% of CD44^+^/CD24^-/low ^cells. Thus, enrichment in cells with markers consistent with human cancer-initiating cells is preserved in the cell lines derived from the tumor that has features of EMT. In contrast to small CD44+/24^-/low ^population, 50–80% of all cell lines had CD24+ or CD44+/24+ fractions, which makes these markers unlikely to represent a stem cell-enriched population. There have been no previous studies implicating CD44+24^-/low ^population as cancer stem cells in mouse models. Our subsequent studies confirmed stem cell nature of this population by their capacity of reconstituting tumors in mice, drug resistance, and expression of many genes associated with normal stem cell biology [31].

## Discussion

In spite of previously established genomic instability associated with Brca1 deficiency [16,27,32-34], transplantation of original Brca1 mouse mammary tumors into naïve recipients and generation of cell lines from these tumors provides a reliable source of material with relatively stable expression profile. However, due to significant heterogeneity among the original tumors, a panel of individual original tumors will be required for selection of a subset of tumors with appropriate characteristics and correlation with human disease.

Transplantation of multiple mouse mammary tumor models into naïve recipients revealed remarkable stability of the cancer genome in MMTV-PyMT and -Wnt1 mammary models [22,35]. Similarly, transplantation of a Brca1 tumor for 2 subsequent passages preserved the molecular features associated with the tumor of origin. However, we found significant differences among the original Brca1 tumors, not previously detected in other mouse mammary models. Thus, in contrast to other mouse mammary tumor models in which the genomic features allow pooling multiple original tumors for expansion *in vivo *to provide virtually unlimited starting material, Brca1 mammary tumors should be analyzed individually and not pooled. Previous studies found significant discrepancies between human disease and some mouse Brca1 deficient tumor models. These differences are likely to reflect the innate heterogeneity of Brca1 mouse mammary tumors and analysis of multiple individual tumors would be required for selection of appropriate tumors that correlate with human disease [28]. In addition, human BRCA1-associated breast and ovarian cancers are multifocal and frequently arise in the contralateral breast. Future studies need to be performed to determine whether differences in gene expression at multiple sites correlate with the heterogeneity found in the mouse models and whether these differences correspond to mixed responses to therapy.

We have previously described that MMTV-PyMT mouse mammary tumors arise earlier in anterior mammary gland, have accelerated tumor growth and can be separated from posterior tumors by gene expression [22]. Our current results pose the intriguing possibility that anterior mammary tumors, such as the original 0_A1 tumor, may be enriched in cancer stem cells. Previous studies indicated that normal CD24^low ^mammary cells are myoepithelial and have high mammary fat pad reconstitution capacity, whereas CD24^+ ^population have low capacity for reconstitution and thus are devoid of normal mammary stem cells [30]. In addition, a recent report showed that freshly isolated normal mammary CD24+ cells have the capacity to form mammospheres. However, when these cells are briefly cultured, the CD24^- ^population becomes enriched in mammosphere-forming and mammary-gland repopulating cells, indicative of a switch in stem cell population [36]. In our studies, the cell lines derived from the original 0_A1 mammary tumor, but not cell lines from posterior tumors contained a population of CD44^+^/CD24^-/low ^cells. We recently confirmed that cells expressing these markers define a cancer stem cell in *Brca1 *deficient cell lines [31]. Analysis of additional markers that distinguish the anterior and posterior *Brca1 *tumors, and their capacity for tumor reconstitution need to be performed to test this hypothesis.

Although transplantation preserves gene expression profile of the original Brca1 deficient tumor, it offers only a limited potential for tumor expansion for further studies since the starting material is a single mouse tumor. Analysis of multiple cell lines derived from each one of the original *Brca1 *tumors revealed additional characteristics of these tumors. In spite of changes consistent with exposure to tissue culture conditions and adaptation to growth as monolayer, such as further loss of basal marker, Cytokeratin 5 [37], and gain in expression of Pdgfr α and β, each group of cell lines segregated with their own tumor of origin. Cell lines derived from a tumor with EMT features, 0_A1, lost expression of keratins, and contained a distinct subpopulation of breast cancer stem cells that express CD44+/CD24- markers [31]. Loss of keratin expression has been correlated with poor prognosis in human breast cancer [38]. To our knowledge, the correlation between enrichment in breast cancer stem cells and EMT features has not previously been reported. It would be important to determine whether other BRCA1-deficient human and mouse tumors with EMT features are enriched in putative breast cancer stem cells.

## Conclusion

Genetic instability associated with BRCA1 deficiency [28,33,34] is believed to be responsible for therapeutic failures, but the role of breast cancer stem cells in tumor progression and drug resistance in this disease are not known. Heterogeneity of gene expression in tumors originating from the same *Brca1 *genetic lesion indicates that these tumors may have distinct biology and respond differently to therapies. Serially transplanted tumors and cell lines derived from these tumors recapitulate the gene expression pattern of the tumor of origin and provide potentially useful tool for preclinical studies *in vitro *and *in vivo*, and studies of Brca1-associted cancer stem cells.

## Competing interests

The author(s) declare that they have no competing interests.

## Authors' contributions

MHW generated cell lines from tumors, characterized them by flow cytometry and western blot, genotyped and isolated RNA from tumors and cell lines, and drafted the manuscript. AIR contributed to the analysis and interpretation of microarray data and helped to draft the manuscript. JIH performed mRNA microarray hybridization, immunohistochemistry and contributed to the analysis and interpretation of microarray data. AIR and JIH contributed equally to the manuscript. MGH performed serial transplantation of tumor suspensions. MRA performed pathological assessment of tumor specimens. CMP supervised microarray analysis, data interpretation and critical review of the manuscript. LV designed the study, participated in data interpretation and manuscript preparation. All authors read and approved the final manuscript.

## Disclaimer

The content of this publication does not necessarily reflect the views or policies of the Department of Health and Human Services, nor does mention of trade names, commercial products, or organization imply endorsement by the U.S. Government.

## Supplementary Material

Additional file 1 Overall similarity of MMTV-driven tumor models. **(A) **Pearson correlation coefficient matrix for pairwise comparisons of log-intensity values among four spontaneous MMTV-PyMT tumors, based on 18,882 probes with reported values in at least 75% of samples (missing-value filter).**(B) **Pearson correlation coefficient matrix for pairwise comparisons of log-intensity values among four spontaneous MMTV-wnt1 tumors, based on 21,860 probes with reported values in at least 75% of samples (missing-value filter). Click here for file

Additional file 2 Heterogeneity of *Brca1 *tumors. **(A) **H&E staining of original *Brca1 *tumors shows three representative types of morphology. A1 tumor shows features of adenosquamous carcinoma composed of sheets of squamous epithelial cells with eosinophilic cytoplasm and large leptochromatic nuclei. Some cells also form clusters of irregular glands. Several areas have whorls and clusters of spindle-shaped tumor cells suggestive of EMT. RP tumor shows features of, glandular carcinoma with no evidence of EMT. RA tumor has features of squamous nonkeratinizing Carcinoma. This tumor also does not show evidence of EMT. **(B) **Immunofluorescent detection of vimentin (red) and SMA (green) (right panels) shows mesenchymal features (vimentin) in tumor 0_A1. SMA stains fibroblasts only and not tumor cells, and is used here as control. Click here for file

Additional file 3 List of most variable genes among mouse models with Brca1 deficiency. List of 339 probes that passed intensity and missing-value filters and had Standard Deviation (SD Gene Vector) >= 1.5 (to remove genes that have standard deviations of observed values less than 1.5, thus selecting for more variable genes across experiments), among 22 arrays encompassing the 5 original *Brca1 *tumors from our study, with 7 tumors from *Brca1*^+/-^, *p53*^+/- ^irradiated (IR, Kohler) mice, and 10 tumors from *Brca1*^*Co*/*Co*^, *p53*^+/-^, *MMTV-Cre *(Furth) from a previous study [17]. Expression ratios for individual tumors are listed. Click here for file

Additional file 4 List of most variable genes among original and passaged *Brca1 *mammary tumors. List of 416 probes that in addition to passing intensity and missing-value filters had a minimum 2.5-fold expression change in either direction from the median value in at least 20% of samples, encompassing 5 original independent *Brca1 *mammary tumors, 4 first-passage and 3 second-passage tumors. Expression ratios for original tumor 0_A1 and the average of expression ratios for transplanted tumors, as well as their fold differences are listed. Click here for file

Additional file 5 Unsupervised Hierarchical Cluster. Unsupervised Hierarchical Cluster of genes and samples using 416 most variable genes across original and transplanted tumors (Additional file 8). Expression data from normal virgin mammary glands dissected from C57Bl6 and SCID mice were included in the analysis to define the contribution of genetic background to the differential gene expression. In the vertical axis, genes were clustered according to similarities in relative expression ratios. Gene clusters associated with genetic background are detailed on the side. Click here for file

Additional file 6 Genotyping of *Brca1 *cell lines by PCR. Photographs of ethidium bromide-stained 1% agarose gels showing the products of PCR genotyping for p53 **(A) **and the *Brca1*^*null *^allele **(B) **using genomic DNA from the indicated cell lines. L, molecular weight marker. C, p53 positive control. All cell lines used in this study were tested and showed loss of p53 allele and presence of the *Brca1*^*null *^allele. Click here for file

Additional file 7 Characterization of *Brca1 *cell lines by Western blot. Analysis of protein expression in whole cell lysates from 16 Brca1 cell lines using antibodies for Cytokeratin 5 (K5), Cytokeratin 18 (K18), c-kit (CD117), Vimentin, and Platelet-derived growth factor receptor alpha (Pdgfrα). Click here for file

Additional file 8 Characterization of *Brca1 *cell lines by flow cytometry. Cell lines derived from anterior (A1.1 and A1.8) or posterior (P2.1 and P3.17) tumors were stained with fluorescently-conjugated antibodies against Cytokeratin 5 **(A) **or CD117 **(B)**, in parallel with isotype control and analyzed by flow cytometry. In the histograms shown, the thick black line represents positive staining and the gray-filled lines represent the isotype control-matched mAb. Click here for file
