# Supplementary figures and images for: Molecular analysis reveals heterogeneity of mouse mammary tumors conditionally mutant for Brca1
Source: Mol Cancer. 2008 Apr 7;7:29. doi: 10.1186/1476-4598-7-29 (PMC2329667; doi:10.1186/1476-4598-7-29)

## Slide 1
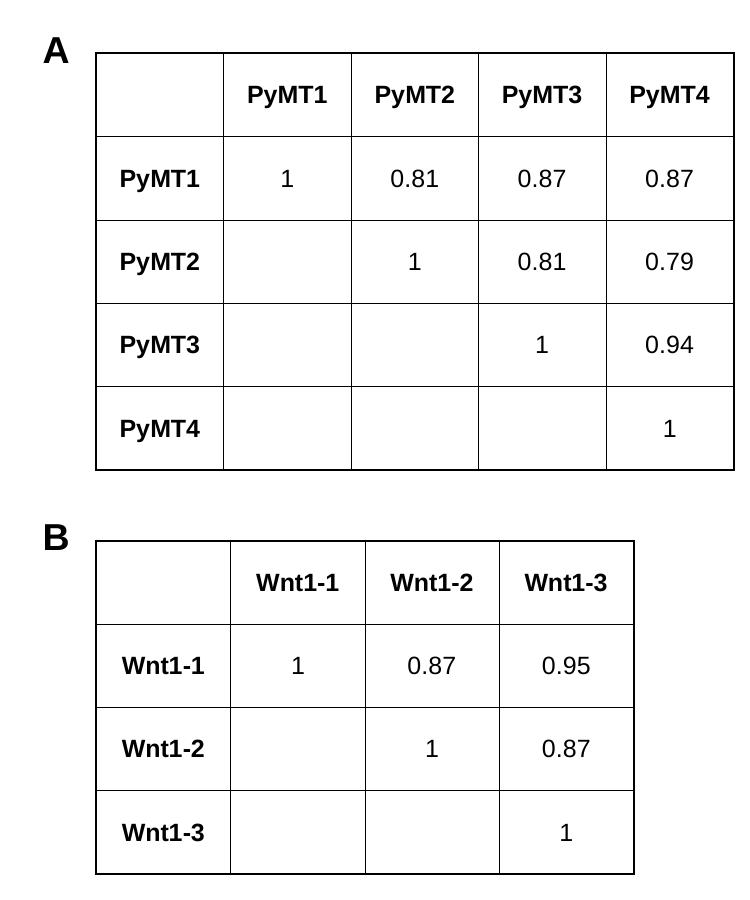

A
| | PyMT1 | PyMT2 | PyMT3 | PyMT4 |
| --- | --- | --- | --- | --- |
| PyMT1 | 1 | 0.81 | 0.87 | 0.87 |
| PyMT2 | | 1 | 0.81 | 0.79 |
| PyMT3 | | | 1 | 0.94 |
| PyMT4 | | | | 1 |
B
| | Wnt1-1 | Wnt1-2 | Wnt1-3 |
| --- | --- | --- | --- |
| Wnt1-1 | 1 | 0.87 | 0.95 |
| Wnt1-2 | | 1 | 0.87 |
| Wnt1-3 | | | 1 |

Supplement: Additional file 1 — Overall similarity of MMTV-driven tumor models. (A) Pearson correlation coefficient matrix for pairwise comparisons of log-intensity values among four spontaneous MMTV-PyMT tumors, based on 18,882 probes with reported values in at least 75% of samples (missing-value filter).(B) Pearson correlation coefficient matrix for pairwise comparisons of log-intensity values among four spontaneous MMTV-wnt1 tumors, based on 21,860 probes with reported values in at least 75% of samples (missing-value filter). [file 1476-4598-7-29-S1.ppt]

## Slide 1
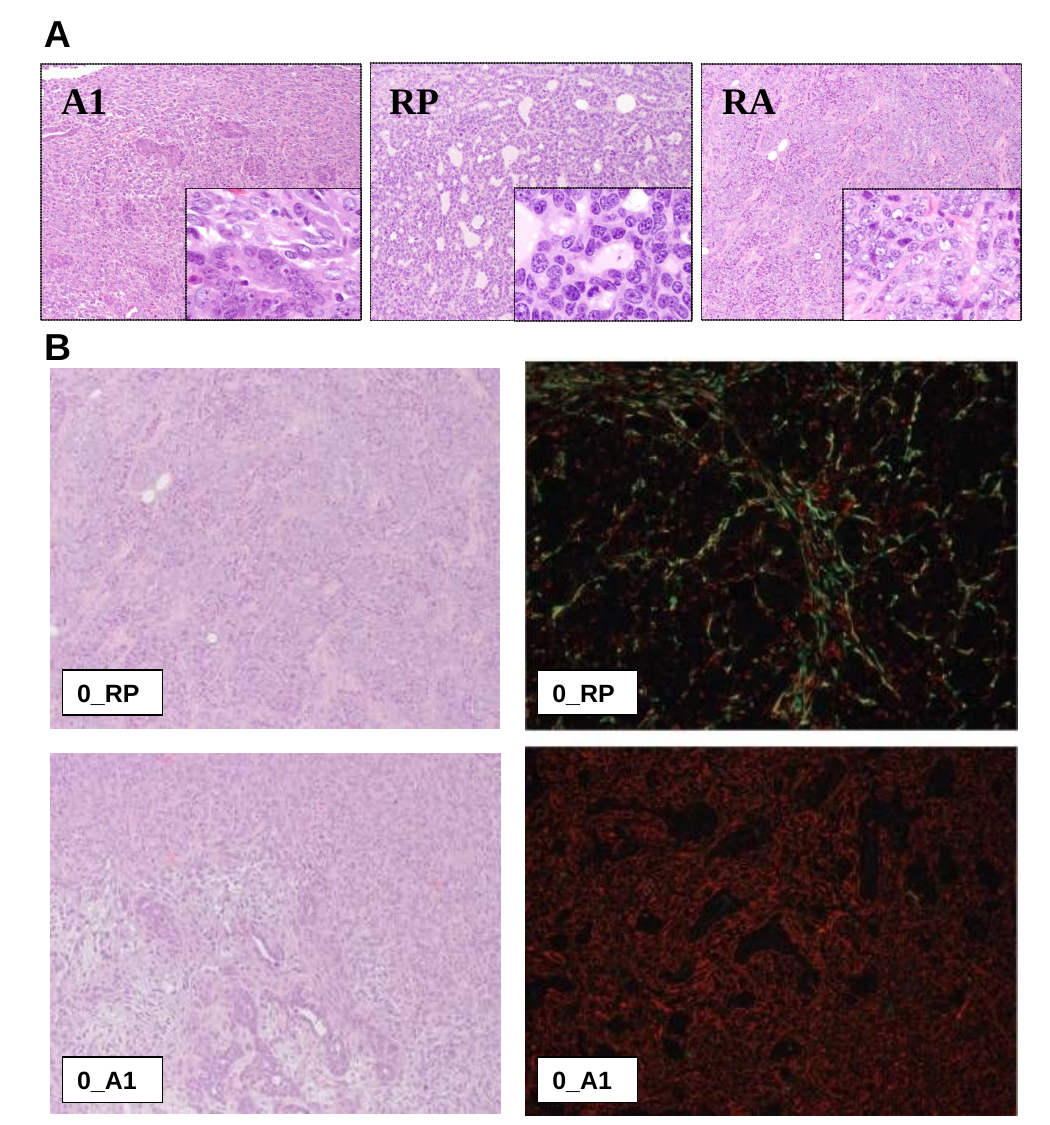

A
A1
RP
RA
B
0_RP
0_RP
0_A1
0_A1

Supplement: Additional file 2 — Heterogeneity of Brca1 tumors. (A) H&E staining of original Brca1 tumors shows three representative types of morphology. A1 tumor shows features of adenosquamous carcinoma composed of sheets of squamous epithelial cells with eosinophilic cytoplasm and large leptochromatic nuclei. Some cells also form clusters of irregular glands. Several areas have whorls and clusters of spindle-shaped tumor cells suggestive of EMT. RP tumor shows features of, glandular carcinoma with no evidence of EMT. RA tumor has features of squamous nonkeratinizing Carcinoma. This tumor also does not show evidence of EMT. (B) Immunofluorescent detection of vimentin (red) and SMA (green) (right panels) shows mesenchymal features (vimentin) in tumor 0_A1. SMA stains fibroblasts only and not tumor cells, and is used here as control. [file 1476-4598-7-29-S2.ppt]

## Slide 1
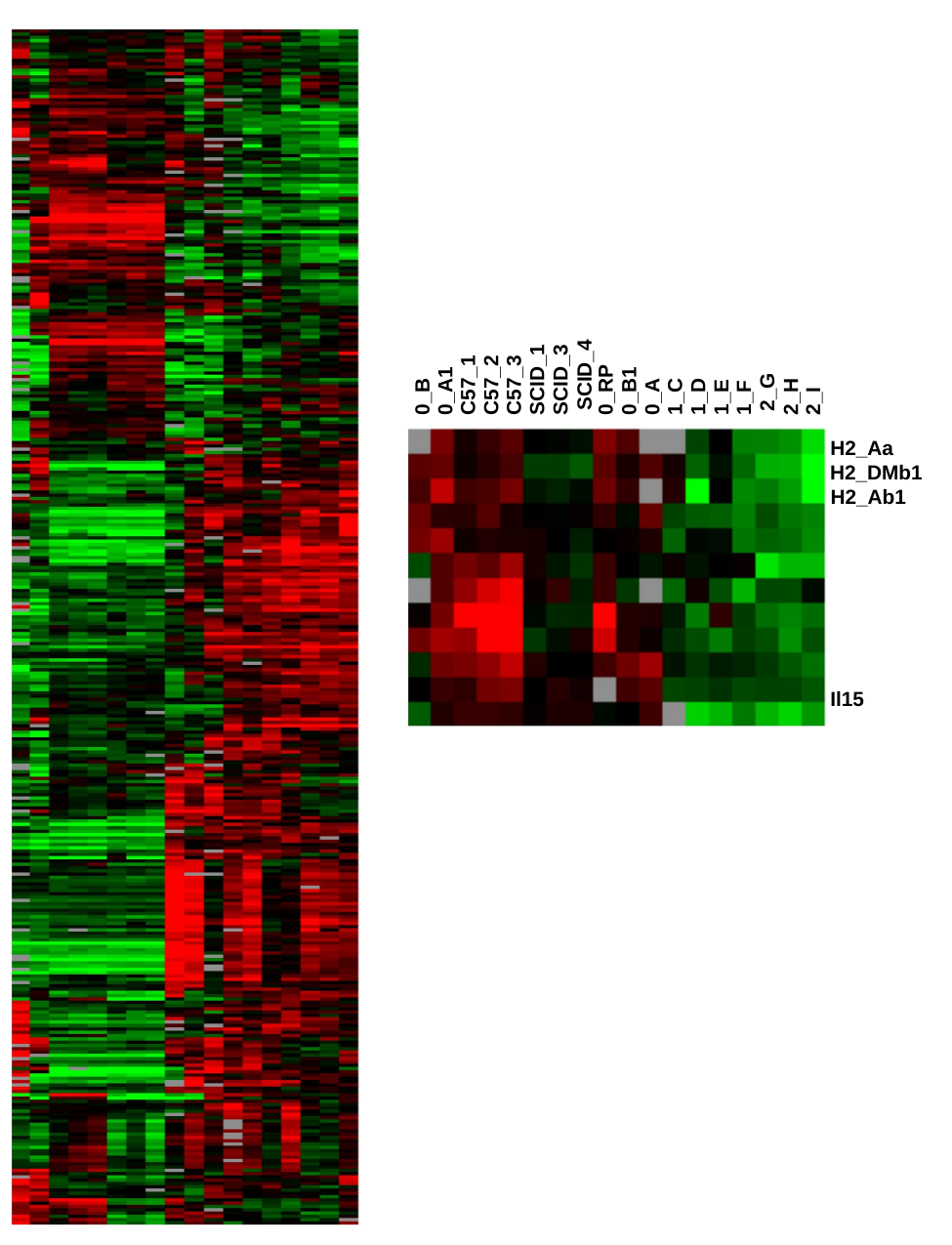

SCID_4
SCID_1
SCID_3
C57_1
C57_2
C57_3
0_RP
0_A1
0_B1
2_G
0_B
2_H
1_F
0_A
1_C
1_D
1_E
2_I
H2_Aa
H2_DMb1
H2_Ab1
Il15

Supplement: Additional file 5 — Unsupervised Hierarchical Cluster. Unsupervised Hierarchical Cluster of genes and samples using 416 most variable genes across original and transplanted tumors (Additional file 8). Expression data from normal virgin mammary glands dissected from C57Bl6 and SCID mice were included in the analysis to define the contribution of genetic background to the differential gene expression. In the vertical axis, genes were clustered according to similarities in relative expression ratios. Gene clusters associated with genetic background are detailed on the side. [file 1476-4598-7-29-S5.ppt]

## Slide 1
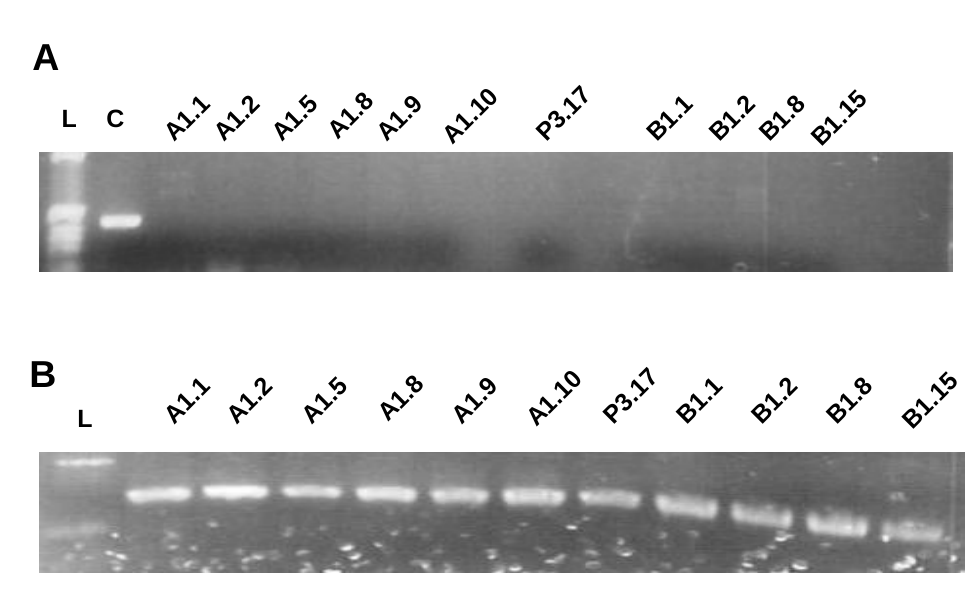

A
P3.17
B1.15
A1.1
A1.9
B1.2
A1.2
A1.5
 A1.8
 A1.10
B1.1
B1.8
 L
C
B
P3.17
B1.15
A1.1
A1.2
A1.5
 A1.8
A1.9
B1.1
B1.2
B1.8
 A1.10
 L

Supplement: Additional file 6 — Genotyping of Brca1 cell lines by PCR. Photographs of ethidium bromide-stained 1% agarose gels showing the products of PCR genotyping for p53 (A) and the Brca1null allele (B) using genomic DNA from the indicated cell lines. L, molecular weight marker. C, p53 positive control. All cell lines used in this study were tested and showed loss of p53 allele and presence of the Brca1null allele. [file 1476-4598-7-29-S6.ppt]

## Slide 1
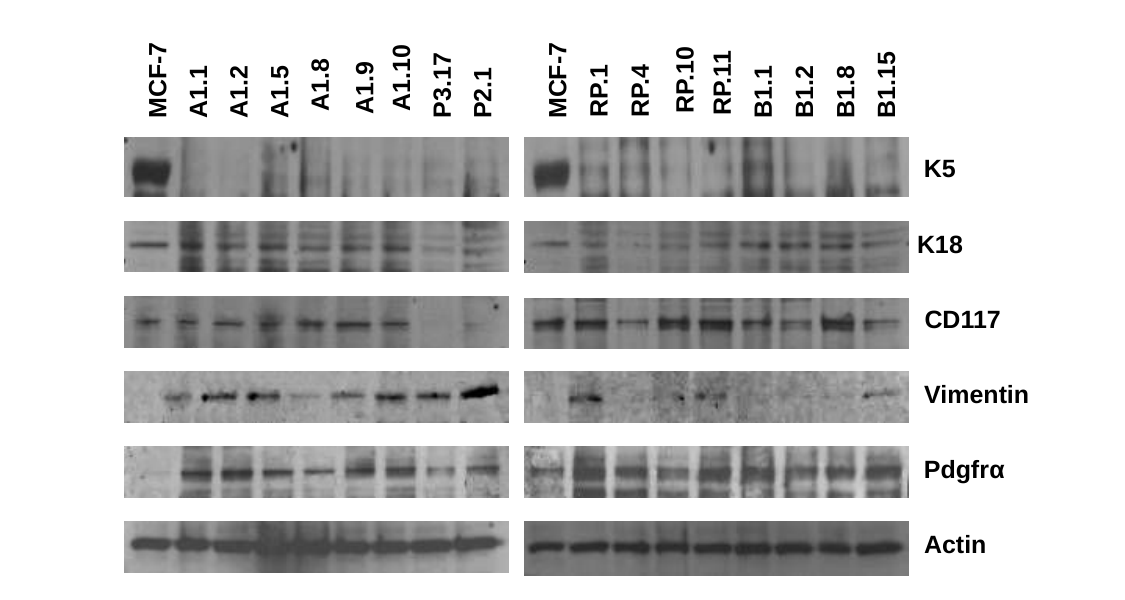

RP.10
MCF-7
MCF-7
 A1.10
RP.11
B1.15
P3.17
A1.9
 A1.8
RP.1
RP.4
A1.1
A1.2
A1.5
B1.1
B1.2
B1.8
P2.1
 K5
K18
 CD117
 Vimentin
 Pdgfrα
 Actin

Supplement: Additional file 7 — Characterization of Brca1 cell lines by Western blot. Analysis of protein expression in whole cell lysates from 16 Brca1 cell lines using antibodies for Cytokeratin 5 (K5), Cytokeratin 18 (K18), c-kit (CD117), Vimentin, and Platelet-derived growth factor receptor alpha (Pdgfrα). [file 1476-4598-7-29-S7.ppt]

## Slide 1
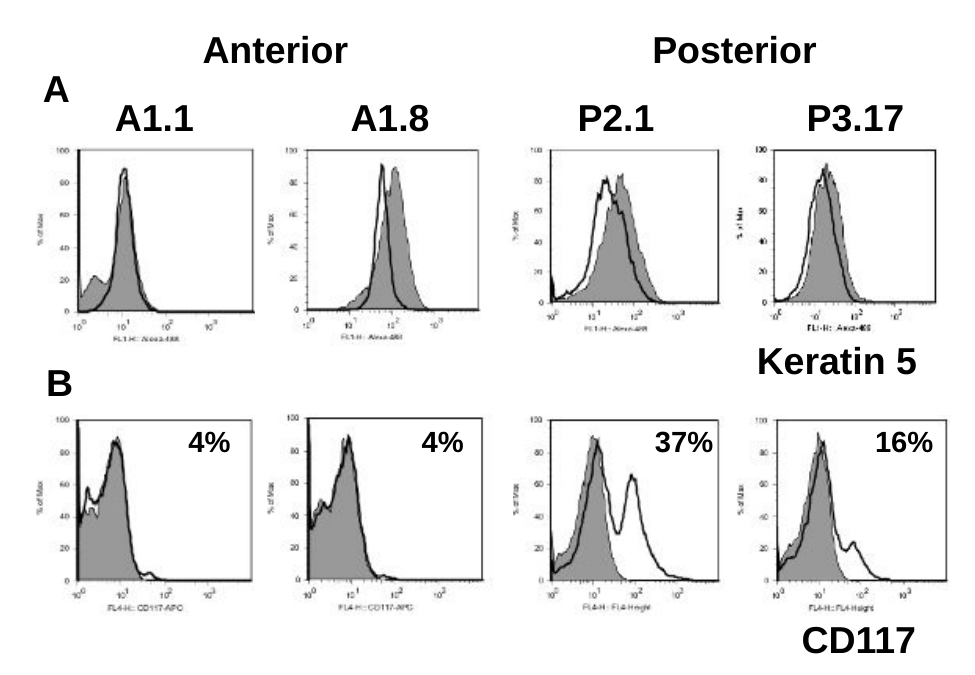

Anterior
Posterior
A
A1.1
A1.8
P2.1
P3.17
 Keratin 5
B
4%
4%
37%
16%
 CD117

Supplement: Additional file 8 — Characterization of Brca1 cell lines by flow cytometry. Cell lines derived from anterior (A1.1 and A1.8) or posterior (P2.1 and P3.17) tumors were stained with fluorescently-conjugated antibodies against Cytokeratin 5 (A) or CD117 (B), in parallel with isotype control and analyzed by flow cytometry. In the histograms shown, the thick black line represents positive staining and the gray-filled lines represent the isotype control-matched mAb. [file 1476-4598-7-29-S8.ppt]
